# The Use of an Invasive Species Habitat by a Small Folivorous Primate: Implications for Lemur Conservation in Madagascar

**DOI:** 10.1371/journal.pone.0140981

**Published:** 2015-11-04

**Authors:** Timothy M. Eppley, Giuseppe Donati, Jean-Baptiste Ramanamanjato, Faly Randriatafika, Laza N. Andriamandimbiarisoa, David Rabehevitra, Robertin Ravelomanantsoa, Jörg U. Ganzhorn

**Affiliations:** 1 Biozentrum Grindel, Department of Animal Ecology and Conservation, University of Hamburg, Hamburg, Germany; 2 Nocturnal Primate Research Group, Department of Social Sciences, Oxford Brookes University, Oxford, United Kingdom; 3 QIT Madagascar Minerals, Rio Tinto, Tolagnaro, Madagascar; Texas A&M University, UNITED STATES

## Abstract

The lemurs of Madagascar are among the most threatened mammalian taxa in the world, with habitat loss due to shifting cultivation and timber harvest heavily contributing to their precarious state. Deforestation often leads to fragmentation, resulting in mixed-habitat matrices throughout a landscape where disturbed areas are prone to invasion by exotic plants. Our study site, the Mandena littoral forest (southeast Madagascar), is a matrix of littoral forest, littoral swamp, and *Melaleuca* swamp habitats. Here, *Melaleuca quinquenervia* has invaded the wetland ecosystem, creating a mono-dominant habitat that currently provides the only potential habitat corridor between forest fragments. We sought to understand the role of this invasive *Melaleuca* swamp on the behavioral ecology of a threatened, small-bodied folivore, the southern bamboo lemur (*Hapalemur meridionalis*). We collected botanical and behavioral data on four groups of *H*. *meridionalis* between January and December 2013. Our results confirm *Melaleuca* swamp as an important part of their home range: while lemurs seasonally limited activities to certain habitats, all groups were capable of utilizing this invasive habitat for feeding and resting. Furthermore, the fact that *Hapalemur* use an invasive plant species as a dispersal corridor increases our knowledge of their ecological flexibility, and may be useful in the conservation management of remaining threatened populations.

## Introduction

Deforestation within the tropics is one of the primary threats to global biodiversity [1, 2]. In addition to forest reduction, fragmentation results in extended edges that are often considered entirely distinct ecosystems from forest interiors [3]. Though fragments may persist after deforestation, most are unsuitable habitat for forest species [4, 5]. Within Madagascar, more than 80% of forest areas exist less than 1 km from an edge [6], thus fragmentation is of great concern for the survival of forest fauna and flora species [7, 8]. Decreasing deforestation rates and reforesting fragmented landscapes would help prevent extinctions [9].

Generally, corridors are defined as thin strips of habitat that connect two or more isolated forest fragments, and many studies validate their utilization by organisms (reviewed in [10; 11]). Although fragmentation of populations may result in genetic erosion and increase extinction risk [12], it has been shown that a mosaic of small, suitable habitat fragments may mitigate these negative effects by acting as a single large habitat if the fragments are linked via corridors [13–17]. A network of forest fragments linked by corridors allowing species to disperse may act as a means to maintain biodiversity and ecological processes in anthropogenic landscapes [18, 19].

The primates of Madagascar are the most threatened mammalian taxon in the world [20]. Their survival is continually jeopardized by hunting for bushmeat, as well as habitat loss due to shifting cultivation and timber harvest [20, 21]. As habitat destruction continues to isolate the remaining lemurs in forest fragments, the need for regenerating forests and connecting those remaining fragments is crucial. As such, it is imperative to understand the responses of native plants and animals to disturbance if we are to create effective buffer zones and corridors that combine secondary and natural habitats [7, 22–24].

It is often thought that invasions by exotic species present a critical hindrance to the preservation of endemic biodiversity, as well as ecosystem restoration efforts [25, 26]. In southeast Madagascar, the Mandena littoral forest matrix exists within a seasonally-inundated flood plain that consists of natural littoral swamp and *mahampy* (*Lepironia mucronata*) wetlands, a portion of which remains inundated throughout the year. It is here, and in similarly inundated areas [27], that the broad-leaved paperbark tree *Melaleuca quinquenervia* (Family Myrtaceae), native to Australia, has been an aggressive disperser [28]. These littoral forests and their animal communities are among the most threatened ecosystems in Madagascar [29, 30]. While the viability of non-native tree plantations has been examined to potentially assist in dispersal and fulfilment of partial habitat requirements for the conservation of lemurs [31–33], the role of introduced and possibly invasive tree species has only been minimally examined [34–36]. This is of considerable interest as littoral forest fragments represent critical refuges for the survival and maintenance of biodiversity in the extremes of climatic variability [37]. Riparian habitats often serve as corridors for multiple taxa [38]; therefore, the conservation of these areas and nearby isolated forest blocks is critical to maintaining resilience.

In view of this conflicting situation, we sought to understand the role of an invasive species habitat (*Melaleuca* swamp) on the behavioral ecology of a small-bodied folivore, the southern bamboo lemur (*Hapalemur meridionalis*) in littoral forest fragments of extreme southeastern Madagascar. Growing knowledge of the ecological flexibility of bamboo lemurs [39–41] makes this species an excellent model with which to examine its ability to utilize distinct habitats, and potentially corridors, within the anthropogenic landscape. Here, we investigated whether *Melaleuca* swamp facilitates movement of *H*. *meridionalis* between littoral forest fragments and/or natural littoral swamp, and whether this invasive habitat provides additional services, e.g., suitable feeding and resting locations.

## Materials and Methods

### Ethics Statement

This study was conducted under the Accord de Collaboration between the University of Antananarivo and the University of Hamburg. Research protocols were approved and permits authorized by Commission Tripartite of the Direction des Eaux et Forêts de Madagascar (Autorisation de recherché n.240/12/MEF/SG/DGF/DCB.SAP/SCB du 17/09/2012), adhering to the legal requirements of Madagascar. We captured adults via Telinject® blow darts (administered by an experienced Malagasy technician) containing a hypnotic anesthesia (4 mg/kg of ketamine hydrochloride or tiletamine hydrochloride), so that the animals neither suffered nor recalled the capturing process. All animals recovered from anesthesia within 1.5 hours at the capture site, and there were no injuries as a consequence of capture and animals were followed until regaining full mobility. This process was repeated at the end of the study in December 2013 to remove the radio-collars from the bamboo lemurs.

### Study Site and Subjects

**Fig 1:**
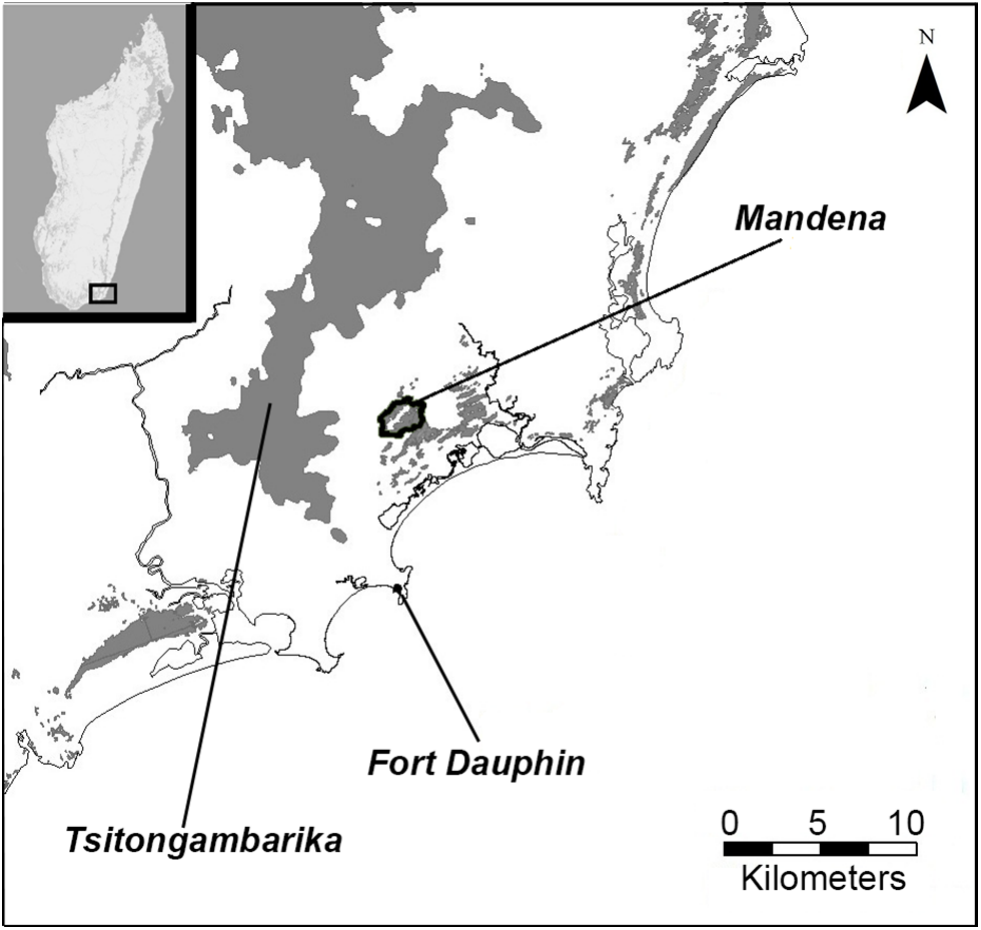
Location of Mandena Conservation Zone in southeast Madagascar, relative to the larger, continuous forest of Tsitongambarika.

Our study was conducted in the Mandena Conservation Zone (24°95’S, 46°99’E) in southeast Madagascar (Fig 1), a protected area approximately 10 km north of Fort-Dauphin (Tolagnaro). This area consists of 148 ha of fragmented and degraded littoral forest, which is characterized as occurring within 3 km of the coast and growing on sandy substrates with a typically low canopy [42], and approximately 82 ha of interspersed natural littoral swamp and invasive *Melaleuca* swamp that separates the two littoral forest fragments [41].

**Fig 2:**
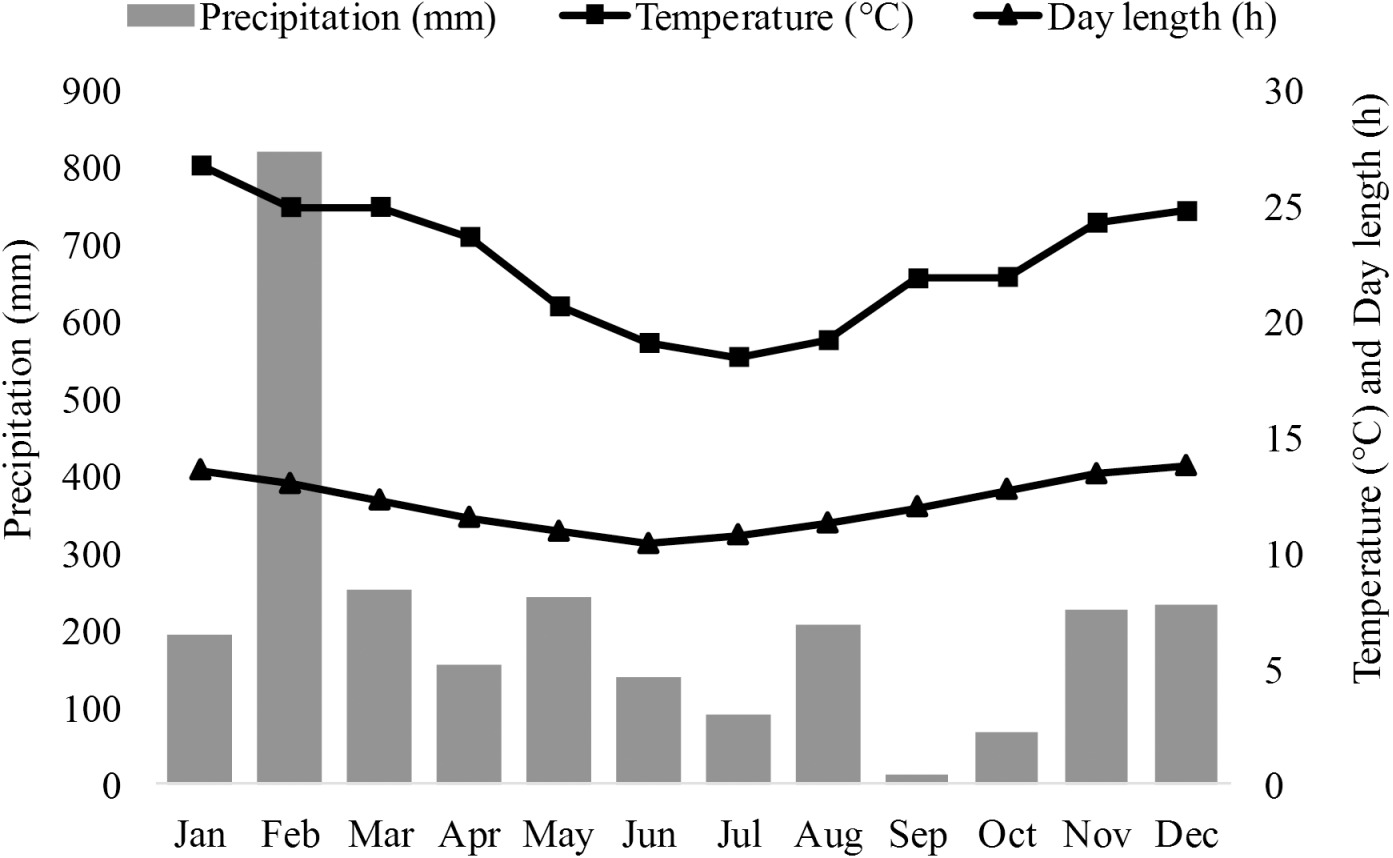
Monthly total precipitation (mm), mean temperature (°C), and mean day length (h) at Mandena in 2013.

During our study period, temperature (°C) was recorded in 30-mins intervals using Lascar EL-USB-1 data loggers, operated by custom software (EasyLog USB Version 5.45, Lascar Electronics). Precipitation (mm) was measured daily at 6:00h using a rain gauge placed within the study site (Fig 2). Day length (a proxy for season) was calculated as the time between sunrise and sunset, as obtained from the US Naval Observatory Astronomical Calendar (http://aa.usno.navy.mil/data), using geographic coordinates for Mandena.

Southern bamboo lemurs (*Hapalemur meridionalis*) are relatively small-bodied folivorous primates with a mean body mass of 1.072 ± 0.107 kg (*X* ± SD; *N* = 15) [41, 43] that maintain a cathemeral activity pattern [44]. This species lives in small social groups with one or two breeding females and typically one breeding male. Within Mandena, *H*. *meridionalis* groups average 5.6 ± 1.5 individuals (*X* ± SD; *N* = 5). In addition to southern bamboo lemur, the cathemeral collared brown lemur (*Eulemur collaris*) and nocturnal gray mouse lemur (*Microcebus murinus*), eastern fat-tailed dwarf lemur (*Cheirogaleus medius*), greater dwarf lemur (*C*. *major*), and southern woolly lemur (*Avahi meridionalis*) are present within Mandena.

Ten adult *H*. *meridionalis* across four neighboring social groups were captured and habituated between October and December 2012. Data were recorded from January to December 2013. As bamboo lemurs are highly cryptic, individuals were fitted with external radio-transmitters with an archival tag (ARC400, Advanced Telemetry Systems, Isanti, USA) that allowed us to more easily follow groups.

### Habitat Characterization

To characterize each distinct habitat, we sampled 25 x 100 m^2^ botanical plots, i.e., 10 in both the littoral forest and littoral swamp, and five in the *Melaleuca* swamp, the latter requiring fewer plots due to its floristic homogeneity. Within each plot we included all trees with a diameter at breast height (DBH) ≥ 5 cm, recording the scientific binomial name and family name of each so as to measure tree diversity, in addition to their height (m) and crown volume (m^3^). The latter was estimated as an ellipsoid via the crown height and two crown diameters, i.e., maximum and perpendicular widths. We further conducted vertical-line transects within each plot, so as to detail the structure and canopy cover for each these three habitats [45]. Lastly, we calculated the Shannon index (*H′*) to determine the species diversity of each habitat. The Mandena littoral forest and littoral swamp that our focal *H*. *meridionalis* groups inhabit are legally protected forests; however, much of the *Melaleuca* swamp falls outside of this demarcation. As such, local people access these unprotected areas daily to harvest wood. To measure the degree to which this occurs, we included felled trees (via tree stumps) in our botanical plots. Lastly, it should be noted that in order for lemurs to access the *Melaleuca* swamp around Mandena, they must descend and traverse a barren, sandy area that would make them visually conspicuous to any potential predators as they leave the canopy cover of the littoral forest. To examine these crossing sites, we measured the distance (m) traversed where lemurs accessed the *Melaleuca* swamp.

### Behavioral sampling

From January to December 2013, we conducted full-day focal follows (sunrise to sunset) with the aim of acquiring 50hrs/month per group for three social groups. We identified individuals using radio-tracking tags with unique-colored pendants. We collected behavioral data via instantaneous focal sampling [46] at 5-min intervals on broad-level activities (resting, feeding, moving, social, and other) and noted the habitat (littoral forest, littoral swamp, and *Melaleuca* swamp). In addition, we collected continuous feeding data each time a focal individual fed, recording the specific food item of the species, and duration of consumption measured to the second. All adult individuals in each group were sampled at least once each month. We further noted each occurrence in which the focal animal utilized the *Melaleuca* swamp corridors connecting the littoral forest fragments.

### GIS analysis

We recorded a focal animal’s GPS location in 15-min intervals using a Garmin GPSMAP 62S unit, and noted the specific habitat type. All ranging data were entered into ArcGIS 10.2 (ESRI) using the Geospatial Modelling Environment (GME) spatial ecology interface [47] with R statistical software version 3.1.2 [48]. We determined each group’s territory using a 95% kernel density estimate [49] and further estimated the area (ha) of each habitat type.

### Statistical analyses

To determine whether the characterization metrics of habitats differed, we used Kruskal-Wallis analyses for tree DBH, height, and crown volumes. We performed non-parametric tests as the data were not normally distributed, even after transformations. To determine the influence of habitat on bamboo lemur activities, a two-way repeated measures ANOVA was performed for each habitat, assessing the monthly proportion of broad-level activities (limited to rest, feed, and travel). Each habitat (littoral forest, littoral swamp, and *Melaleuca* swamp) was treated as the within-subjects factor, with groups acting as the between-subjects factor. Additionally, abiotic factors of total precipitation (mm), mean temperature (°C), and mean day length (h) per month were included in the model as covariates. The model errors for the repeated-measures ANOVA (via unstandardized residuals) were found to be normally distributed using the Kolmogorov-Smirnov test, allowing for the continuation of parametric analyses. Adjusted *p*-values are reported according to the Huynh–Feldt correction when assumptions of sphericity were violated; uncorrected biases from lack of sphericity can otherwise inflate *F*-statistics [50]. All analyses were performed using PASW v. 21.0 and significance was set at *p* < 0.05.

## Results

### Habitats

Compared to the botanically diverse littoral forest and littoral swamp, the *Melaleuca* swamp comprised six tree species, each from a distinct family (Table 1). While 90.02% was *M*. *quinquenervia*, the remainder comprised native *Typhondorum lindleyanum* (7.52%), *Pandanus platyphylus* (2.09%), *Barringtonia racemosa* (0.27%), *Ravenala madagascariensis* (0.05%), and exotic *Acacia mangium* (0.05%). Tree analyses found that the three variables were significantly different between habitats (DBH (cm): Kruskal-Wallis *H* = 363.70, *df* = 2, *p* < 0.001; height (m): Kruskal-Wallis *H* = 195.43, *df* = 2, *p* < 0.001; crown volume (m^3^): Kruskal-Wallis *H* = 350.33, *df* = 2, *p* < 0.001).

**Table 1:** Comparison of trees (mean ± SD) measured in different habitats within Mandena.

Habitat	*N*	Species (*N*)	Families (*N*)	DBH (cm)	Height (m)	Crown volume (m^3^)	Shannon (*H*′)
Littoral Forest							
≥ 5 cm (DBH)	1454	84	40	9.53 ± 5.09	7.22 ± 1.48	10.41 ± 18.31	3.54 ± 0.05
Littoral Swamp							
≥ 5 cm (DBH)	2211	49	32	11.66 ± 5.95	6.47 ± 1.13	3.91 ± 6.68	2.92 ± 0.08
*Melaleuca* Swamp							
≥ 5 cm (DBH)	2194	6	6	12.11 ± 5.89	6.76 ± 2.33	4.61 ± 7.64	0.39 ± 0.07

**Fig 3:**
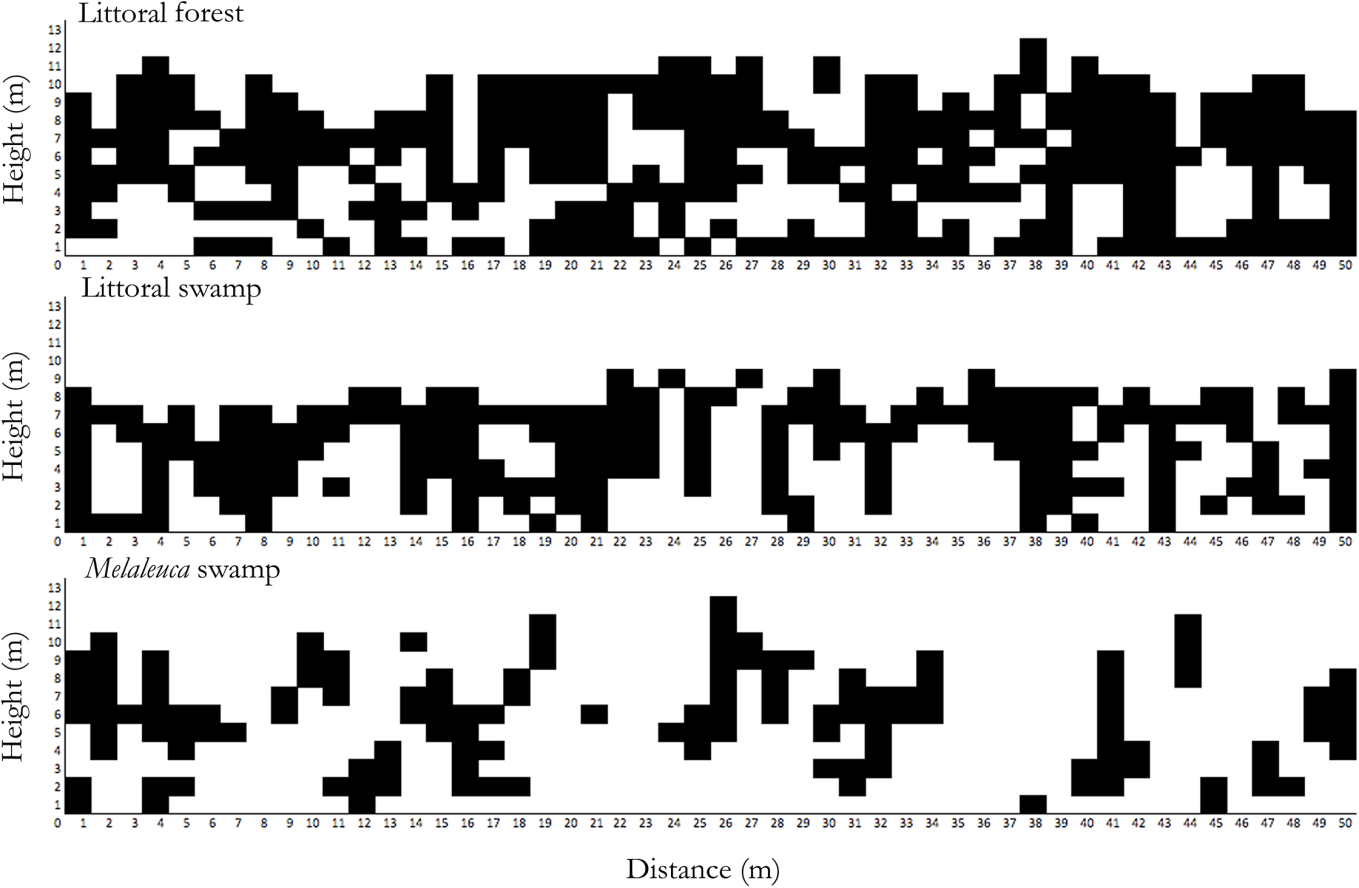
Vertical structure comparison between each of the three Mandena habitats based on Gautier-transects [45]: littoral forest, littoral swamp, and *Melaleuca* swamp.

As a demonstration of human impact on the *Melaleuca* habitat, we recorded 65 *M*. *quinquenervia* with a mean DBH (*X* ± SD) of 12.85 ± 8.89 cm felled within our five *Melaleuca* swamp botanical plots between January and December 2013. In addition to timber harvesting and significantly different tree metrics, habitats were further distinguished by their vertical structure (Fig 3). The mean distance of the eight confirmed crossing sites that *Hapalemur* groups utilized in order to access the *Melaleuca* habitat from adjacent littoral forest is 9.75 ± 2.71 m (*X* ± SD).

### Spatial analysis

**Fig 4:**
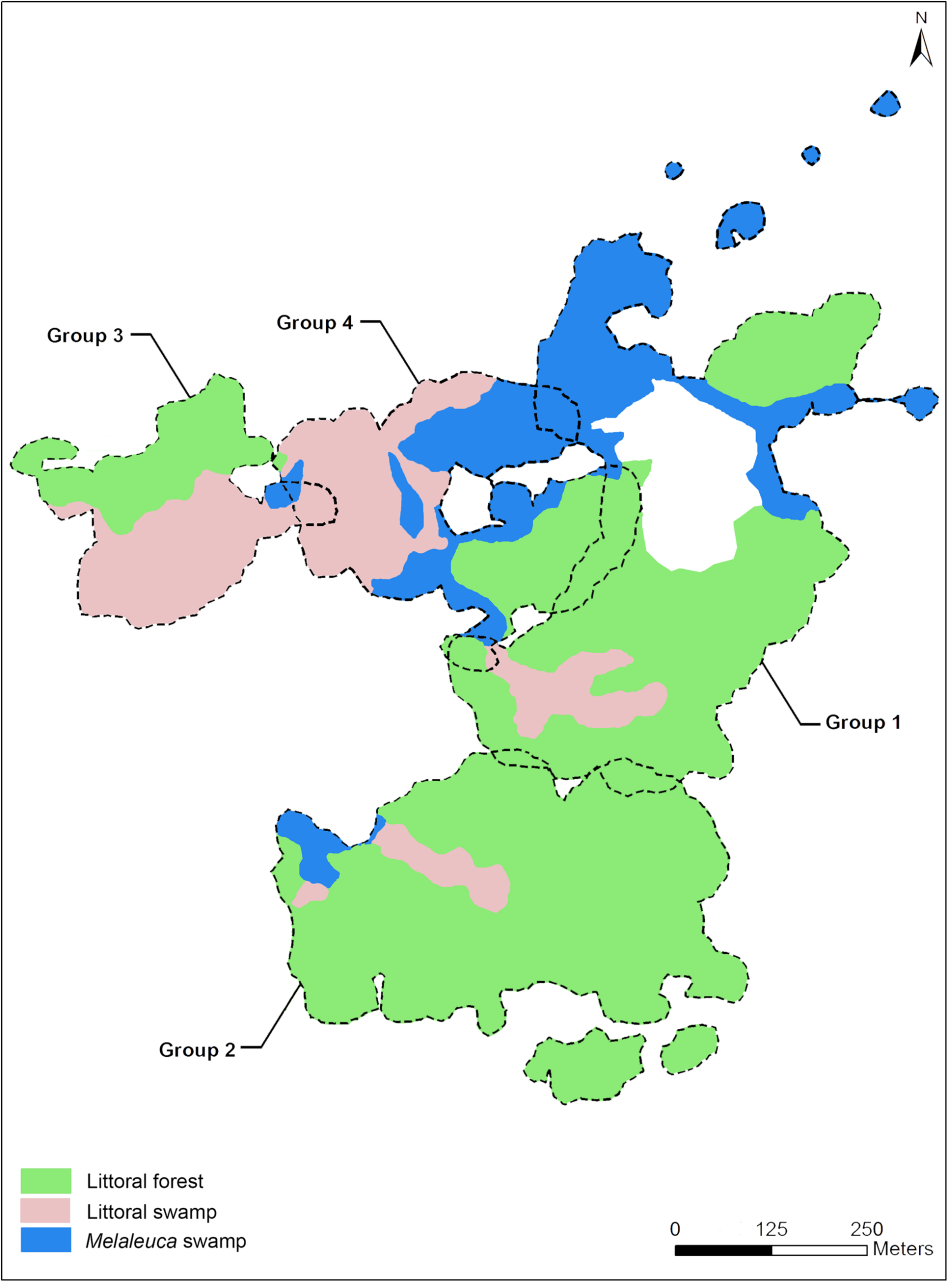
Home ranges (95% kernel) of *Hapalemur meridionalis* focal groups at Mandena between January and December 2013. Areas for each habitat (i.e., littoral forest, littoral swamp, *Melaleuca* swamp) are shown within each.

The total area (ha) of both home ranges utilized by groups 1 and 2 were even in size, while the home range of group 4 was substantially smaller (Table 2). The *Melaleuca* swamp habitat constituted large portions of the home ranges of groups 1 and 4, while it appeared to be minimal for group 2 (Fig 4).

**Table 2:** Total area (ha) and area per habitat type as obtained via 95% kernel density estimate.

Group	Forest (ha)	%	Swamp (ha)	%	*Melaleuca* (ha)	%	Total (ha)
1	11.67	53.45	1.27	5.81	8.89	40.74	21.82
2	19.95	94.16	0.85	4.03	0.38	1.80	21.19
4	3.68	27.13	4.69	34.58	5.19	38.29	13.55

Group 3 habitat data were not included as they constitute a smaller dataset.

### Activity and Habitat

We observed *H*. *meridionalis* for 1,762 hours between January and December 2013 across 194 focal days. Groups differed in the proportion of time (i.e., percentage) spent resting in each habitat (Forest = 27.99 ± 2.21; Swamp = 7.23 ± 1.35; *Melaleuca* = 4.76 ± 1.02 (*X* ± SE; *N* = 36 months)).

There were no differences in overall rates of resting between habitat types (Table 3). Significant interactions were found between temperature and habitat, day length and habitat, as well as group and habitat. Considering the covariates, resting is significantly affected by temperature, but not affected by seasons (i.e., day length). Post-hoc analyses of groups revealed a significant difference between groups 1 and 4 (*p* = 0.004), while groups 1 & 2, and 2 & 4 were similar in the proportion of time and location they chose to rest.

**Table 3:** Repeated measures analysis of variance for effects of habitat type on activity (using monthly percentages) of *H*. *meridionalis* at Mandena, January-December 2013. Significant differences indicated in bold.

Activity	Source of variation	*df*	*F*-ratio	*p*
**Rest**	*Within-subjects*			
	Habitat	1.786	1.303	0.278
	Temperature x Habitat	1.786	3.521	**0.041**
	Precipitation x Habitat	1.786	2.170	0.129
	Day length x Habitat	1.786	3.756	**0.034**
	Group x Habitat	3.573	23.713	**<0.001**
	*Covariates*			
	Temperature	1	7.144	**0.012**
	Precipitation	1	1.330	0.258
	Day length	1	3.271	0.081
	*Between-subjects*			
	Group	2	4.848	**0.015**
**Feed**	*Within-subjects*			
	Habitat	1.995	3.523	**0.036**
	Temperature x Habitat	1.995	3.868	**0.026**
	Precipitation x Habitat	1.995	0.991	0.377
	Day length x Habitat	1.995	6.249	**0.003**
	Group x Habitat	3.990	25.583	**<0.001**
	*Covariates*			
	Temperature	1	24.282	**<0.001**
	Precipitation	1	1.431	0.241
	Day length	1	10.154	**0.003**
	*Between-subjects*			
	Group	2	0.473	0.628
**Travel**	*Within-subjects*			
	Habitat	1.995	1.396	0.256
	Temperature x Habitat	1.995	1.309	0.278
	Precipitation x Habitat	1.995	3.454	**0.038**
	Day length x Habitat	1.995	2.108	0.131
	Group x Habitat	3.991	27.393	**<0.001**
	*Covariates*			
	Temperature	1	15.279	**<0.001**
	Precipitation	1	1.619	0.213
	Day length	1	5.504	**0.026**
	*Between-subjects*			
	Group	2	0.107	0.899

When considering feeding activity, there was no appreciable difference in the mean proportion of time each group fed; however, there were significant differences in the average proportion of feeding between the habitats (Forest = 25.52 ± 2.75; Swamp = 5.72 ± 1.09; *Melaleuca* = 11.35 ± 2.31 (*X* ± SE; *N* = 36)). Significant interactions were revealed between temperature and habitat, day length and habitat, and group and habitat (Table 3). Furthermore, feeding activity is affected by both temperature and day length, varying seasonally. Post-hoc analyses showed no discernible effect of feeding between groups.

Traveling showed no differences in means between the groups (Table 3), while the main effect of habitat was revealed to have no influence (Forest = 6.69 ± 0.51; Swamp = 2.02 ± 0.37; *Melaleuca* = 1.41 ± 0.21 (*X* ± SE; *N* = 36)). There were significant interaction effects between precipitation and habitat, and group and habitat. Considering the covariates, traveling is affected by both temperature and day length, varying seasonally. Post-hoc analyses showed no discernible effect of feeding between groups.

### *Melaleuca* habitat use

**Fig 5:**
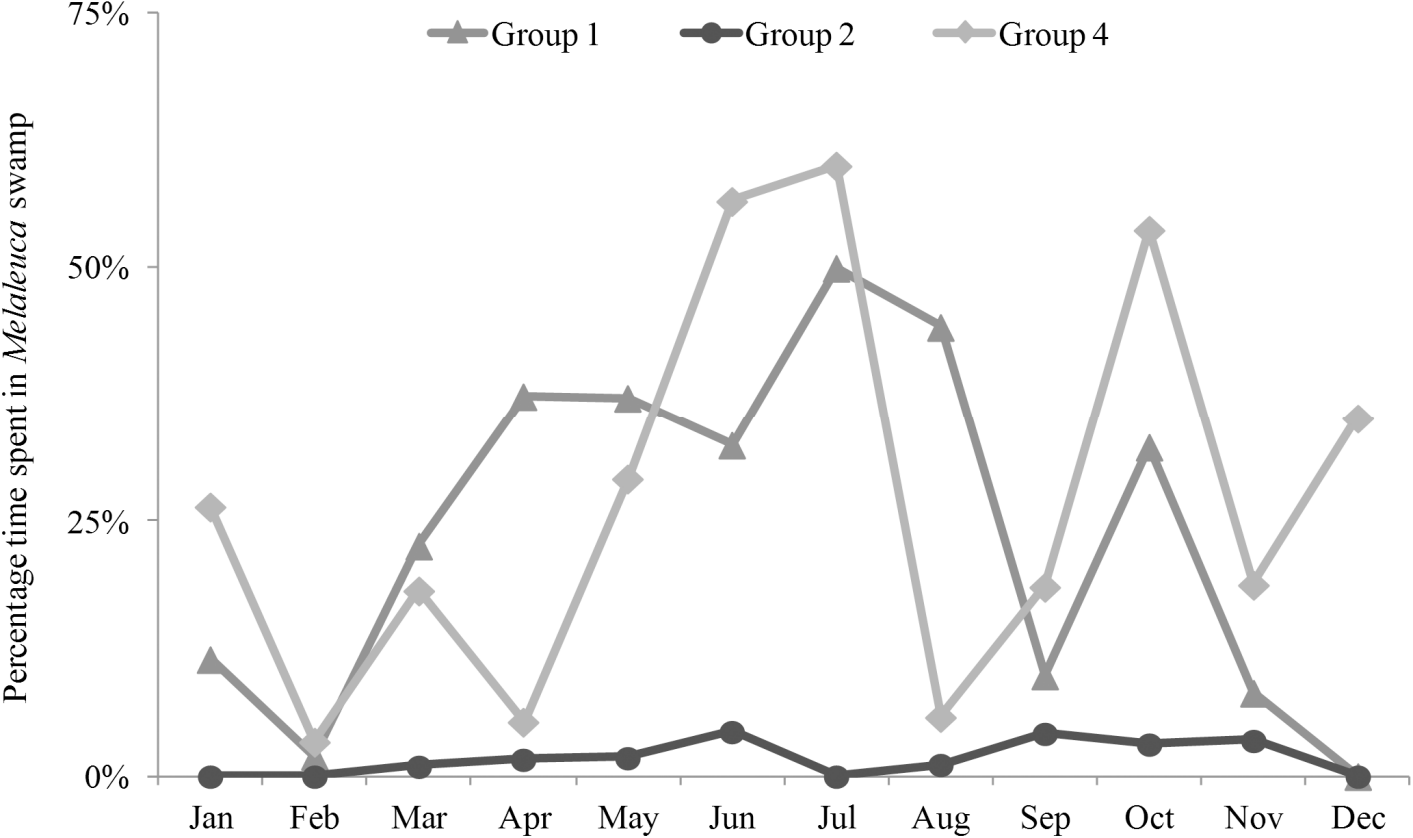
Monthly percentage of time spent by *H*. *meridionalis* groups in the *Melaleuca* habitat from January-December, 2013.

Considering individual focal days, *H*. *meridionalis* were observed to access *Melaleuca* habitat on 54.12% of days, although this only constituted 18.55% of our total observation record (Table 4). Despite this, both groups 1 and 4 accessed this invasive habitat often, while the minimal proportion of *Melaleuca* within the territory of group 2 was still utilized on greater than 20% of observation days. In terms of monthly percentage of time, however, group 2 utilized *Melaleuca* less compared to the other lemur groups (Fig 5).

**Table 4:** Role of *Melaleuca* swamp habitat on daily activity budget of *H*. *meridionalis*.

	Observ. days	Accessed *Melaleuca*		
		Number of days	% of days	% of observation record
Group 1	65	37	56.92	24.22
Group 2	63	13	20.63	1.70
Group 4	66	55	83.33	30.97
**Overall**	**194**	**105**	**54.12**	**18.55**

Two of the three bamboo lemur focal groups fed regularly on the flowers of this invasive species when available. Group 1 was observed to feed on the flowers of *M*. *quinquenervia* for 110.65 mins, constituting 0.79% of the annual diet. While group 2 never fed on *M*. *quinquenervia* flowers, group 4 spent 2.43% of their annual total feeding record (316.32 mins) selecting for them.

## Discussion

Our results show that *H*. *meridionalis* use the introduced stands of *M*. *quinquenervia* substantially. *Melaleuca* and swamp habitats were often inundated by water during the warm/wet austral summer, which may restrict lemur use of these habitats to the cooler/drier months. However, examination of the monthly use of *Melaleuca* habitat by each group shows that while they spend less time here in the warmer months, they are capable of accessing this habitat when inundated. In fact, it is during this inundated period (Oct-Apr) when *M*. *quinquenervia* flowers in short, frequent bursts, but availability of this food item does not appear to influence the proportion of time bamboo lemurs spend in this habitat. This is especially true of groups 1 and 4, which spent considerable time feeding on these flowers when available, something that collared brown lemurs (*E*. *collaris*), eastern fat-tailed dwarf lemurs (*C*. *medius*), and gray mouse lemurs (*M*. *murinus*) have also been observed to exploit [51].

There were larger proportional areas of *Melaleuca* habitat in the territories of groups 1 and 4, thus they spent more time resting, feeding, and travelling in this habitat compared to group 2. Furthermore, these social groups were occasionally found before sunrise sleeping in a *Melaleuca* tree, typically huddled together at an approximate height of 7m. The overall difference in time-budget between the groups (when controlling for the effect of habitat and the covariates) was similar for feeding or travelling activity categories, but displayed appreciable differences for resting. Precipitation was not influential, except in the case of travelling. Additionally, we observed *E*. *collaris*, *M*. *murinus*, and southern woolly lemurs (*A*. *meridionalis*) travelling and sleeping in the *Melaleuca* habitat (see also [35]).

Indigenous and/or exotic tree species can provide benefits to both local people and primates [36, 52, 53]; the presence of *Melaleuca* in Mandena has value as habitat and as timber. Local people have begun to harvest these trees daily with the recent legal protection status of the Mandena littoral forest. Gaps in the *Melaleuca* canopy would allow for continued growth of terrestrial swamp vegetation, specifically graminoid species, which constitute a large portion of the *H*. *meridionalis* diet [41]. *Melaleuca* may have value as a temporary, fast-acting solution to connecting fragments while more long-term conservation solutions are being put in place, e.g., the Mandena nursery/reforestation efforts [35]. In the case of Mandena, the exotic *Melaleuca* acts similarly to a plantation forest for native fauna; while not as ideal as natural littoral forest, it provides valuable habitat and may possibly contribute to the conservation of endemic fauna [54]. Many studies from various countries, including Madagascar, have documented that exotic plantation forests can provide habitat for numerous native forest fauna [31, 55–61]. As an example, threatened bird species such as *Apteryx mantelli*, *Casuarius casuarius*, and *Upupa epops* have been known to occur in substantial populations in some exotic plantation habitats [60, 62, 63] (but see [64, 65]). Furthermore, primates such as black howler monkeys (*Alouatta pigra*) have been reported to thrive in *Eucalyptus* spp. plantations [66], mantled howler monkeys (*A*. *palliata*) are able to use shade-grown coffee (*Coffea arabica*) as the core of their habitat range [67], while siamang (*Hylobates syndactylus*) are known to occur in rubber (*Hevea brasiliensis*) and dammar gum (*Shorea javanica*) tree plantations [68]. As for Malagasy primates, many lemur genera (including *Eulemur*, *Hapalemur*, *Indri*, *Cheirogaleus*, *Microcebus*, and *Lepilemur*) are known to use old growth eucalypt (*Eucalyptus* sp.) plantations [31], while some occasionally utilize and feed from mono-stands of invasive guava (*Psidium* spp.) [39, 69]. Bamboo lemurs (both *Hapalemur* spp. and *Prolemur simus*) similarly use an exotic species habitat and appear to be relatively adaptable within anthropogenic landscapes: they have been seen crop-raiding agricultural fields at some sites and even living in a coffee plantation [39, 40, 70] (but see [71]).

That the lemurs utilized invasive *Melaleuca* for behavioral activities demonstrates its potential role as a riparian corridor to facilitate dispersal. From October 2012 to December 2013 we confirmed three separate *Hapalemur* dispersals that utilized *Melaleuca* corridors to emigrate from their natal group, while a fourth dispersal remains unconfirmed. While our data indicate that *H*. *meridionalis* are tolerant of habitat degradation and fragmentation, habitat matrix composition and connectivity have been shown to influence dispersal in various birds and mammals [72, 73], e.g., hazel grouse *Bonasa bonasia* [74], barred antshrikes *Thamnophilus doliatus* [75], Angola black-and-white colobus *Colobus angolensis palliatus* [76], and various marsupials [77]. Furthermore, exotic tree plantations/forests have been demonstrated to facilitate dispersal for a wide range of taxa [78, 79], for example, dispersal of the chucao tapaculo (*Scelorchilus rubecula*) is facilitated by the vertical structure rather than plant species composition of the corridor, in this case shrub fields dominated by 1–2 m tall invasive *Baccharis magellanica* [80].

While instances of successful dispersal provide a glimmer of hope, the further fragmentation of remaining forests is of great concern if forest species of Madagascar are to persist [8]. Lemurs fulfill important ecological roles, e.g., they are the primary seed dispersers and pollinators, and are essential for maintaining the island’s unique forests; their loss would likely trigger extinction cascades [55, 81]. Although the fate of all lemur species should be considered precarious due to increasing habitat destruction, the knowledge that some lemurs are able to cope with this degradation (to a certain degree) should be seen as positive. Recent studies have begun to alter our view of *Hapalemur* spp. as dietary specialists: they demonstrate dietary flexibility and some populations are able to subsist on items other than bamboo [39, 41, 82]. Some primate species adapted to narrow ecological specializations may be sensitive to natural or anthropogenic habitat perturbations [83, 84], whereas others have been shown to adjust to these changing environments [75, 85].

The ecological flexibility of the southern bamboo lemurs might provide a model for conservation action to help some of their congeners to survive. Among the most threatened within the genus is the Lac Alaotran gentle lemur (*H*. *alaotrensis*), assessed by the IUCN [86] as Critically Endangered (CR B1ab(iii,v)), due to its greatly restricted range that is becoming increasingly populated while the remaining viable habitat continues to shrink [87]. They subsist on a diet limited to sedges and non-bamboo grasses [82], similar to *H*. *meridionalis* when it is in the *Melaleuca* habitat. In captivity, however, *H*. *alaotrensis* regularly display a preference for bamboo [88], suggesting little divergence from congeners, with a flexibility that may allow them to persist in habitats outside of Lac Alaotra. As it would appear that all former subspecies of *H*. *griseus* maintain some dietary plasticity, in that they are not restricted to bamboo forests [39, 41, 82], perhaps conservationists need to rethink their strategy when considering how to save species of the *Hapalemur* genus.

## Supporting Information

S1 Table Monthly means for seasonal variables and broad-level activity categories in different habitats for three *Hapalemur meridionalis* groups at Mandena, Madagascar (2013). (XLSX)
